# Self-Assembly of Block and Graft Copolymers in Organic Solvents: An Overview of Recent Advances

**DOI:** 10.3390/polym10010062

**Published:** 2018-01-11

**Authors:** Leonard Ionut Atanase, Gerard Riess

**Affiliations:** 1 Faculty of Dental Medicine, “Apollonia” University, 700399 Iasi, Romania; 2 Research Institute “Academician Ioan Haulica”, 700399 Iasi, Romania; 3 University of Haute Alsace, Ecole Nationale Supérieure de Chimie de Mulhouse, Laboratoire de Photochimie et d’Ingénierie Macromoléculaires, 68093 Mulhouse CEDEX, France; gerard.riess@uha.fr

**Keywords:** self-assembly, micelle, organic solvents, block copolymers, graft copolymers, triblock terpolymers

## Abstract

This review is an attempt to update the recent advances in the self-assembly of amphiphilic block and graft copolymers. Their micellization behavior is highlighted for linear AB, ABC triblock terpolymers, and graft structures in non-aqueous selective polar and non-polar solvents, including solvent mixtures and ionic liquids. The micellar characteristics, such as particle size, aggregation number, and morphology, are examined as a function of the copolymers’ architecture and molecular characteristics.

## 1. Introduction

The self-assembly and micellization of block and graft copolymers with the formation of structured nanoparticles have attracted a major interest over the last decades. Since the pioneering studies of Tuzar and Kratochvil [1] on micellar systems in organic solvents, one of the major research trends afterward was to develop aqueous-based systems. These systems are of considerable interest in biomedical applications. This field has been quite extensively reviewed by different research groups [2,3,4,5,6]. During the same time period, the research activity on non-aqueous micellar systems was ongoing, but somehow to a minor extent, as outlined by the review articles published at the beginning of this century [7,8,9].

Therefore, the aim of the present review article is to update and highlight the recent advances related to the self-assembly of block and graft copolymers in non-aqueous media. This topic is one of interest not only from a theoretical point of view, but also due to its widespread application possibilities in membrane technology, surface modification of pigments and fillers, non-aqueous dispersion, lubricant additives, etc. In addition to these specific applications, non-aqueous micellar systems may have certain advantages with respect to the aqueous micellar dispersions. In fact, it has to be recalled that the micelle’s characteristics generated by a polyA-*b*-polyB copolymer in the presence of selective aqueous or organic solvent S are depending to a large extent to the Flory-Huggins interaction parameter, such as χ_AB_, χ_AS_ and χ_BS_, which are the polymer/polymer and the polymer/solvent interaction parameters, respectively. These parameters are furthermore directly correlated with the solubility parameter δ of the compounds. In the common practice, with a homologues series of organic solvents, such as alcanes, esters, or alcohols, it will therefore be easier than for pure water to adjust the χ values of the solvent to χ values of the copolymer sequences. Finally, it is quite obvious that the self-assembly of hydrophobic/hydrophobic copolymers, which correspond to a large part of those synthesized up to now, may be achieved only in organic media.

In view of this background, the present literature survey from the last decade, including the authors’ contributions, is structured as follows: (i) a brief section is provided in order to review the experimental techniques; (ii) the self-assembly of linear AB and ABA block copolymers in polar and non-polar solvents, as well as in specific solvent mixtures and in ionic liquids (ILs), are outlined; (iii) a major section is then devoted to ABC linear triblock terpolymers and their specific multi-compartment micellar morphologies; and, (iiii) the micellization aspects of graft copolymers and their possibility of forming unimolecular micelles will be described in the last section.

Out of scope of this review are the following topics: bottle-brush micelles; rod-coil copolymers; interpolymer complexes of block and graft copolymers; and, micellization in super critical carbon dioxide (CO_2_).

## 2. Experimental Section

### 2.1. Synthesis and Molecular Characteristics

It is now accepted that well-defined block and graft copolymers can be obtained by living sequential and controlled radical polymerization techniques [10,11,12,13,14,15,16]. However, one has to bear in mind that even the so-called *well-defined* copolymers may present a non-negligible *polydispersity* in their composition and molecular weight for linear AB, ABA, and ABC block copolymers. The same type of limitation not only affects block copolymers, but graft copolymers as well. In addition, a *polydispersity* in graft density has to be considered for graft copolymers.

From a practical point of view, it is worth noting that AB block copolymers can also be synthesized by the so-called Polymerization Induced Self-Association (PISA) technique, which is carried out essentially in organic solvents. In-situ micellization is directly involved in this synthesis technique, as outlined recently by Armes and coworkers [17,18,19,20,21]. The determination of the copolymers’ molecular characteristics, such as molecular weight, composition, and end-group functionality, is well-documented in recent review articles [10,11,12].

### 2.2. Solvents

It is well-established that the solubility of a given polymer is directly defined by the Flory-Huggins solvent/polymer interaction parameter (χ). However, this parameter is not always available. Therefore, for the selection of a selective solvent, which is required in order to induce the self-assembly of a block or graft copolymer, it is of common practice to use the concept of “*solubility parameter*” (δ) of solvents and polymers. Among the various solubility scales, the Hildebrand δ value is the most frequently used. The δ_t_ value involves the dispersive δ_d_ and polar δ_p_, as well as the hydrogen bonding δ_h_ contributions, such as: δ_t_ = (δ_d_^2^ + δ_p_^2^ + δ_h_^2^)^1/2^. For non-ionic solvents, and particularly for aliphatic hydrocarbon compounds, δ_p_ and δ_h_ are negligible with respect to δ_d_. The δ_t_ values are therefore in the range of 15 to 18 MPa^1/2^. This approach is advantageous for solvent mixtures, often used in micellization studies, because defining an “*average solubility parameter*” is possible. The usual non-aqueous solvents used in the micellization experiments are listed in Table 1, with an indication of their solubility parameter [22].

### 2.3. Self-Assembly Techniques

The self-assembly techniques and the micellar characterization methods used for amphiphilic block and graft copolymers have been described in several review articles [7,8,9,23]. The simplest method for the preparation of micelles is the direct dissolution of copolymer samples in a selective solvent for one of the sequences. It has to be kept in mind that this procedure is recommended only for copolymers with low molecular weights and short insoluble sequences. In another technique, the micellization occurs by the addition of a non-solvent to a common solvent, in which the copolymer is initially molecularly dispersed. However, this technique may induce aggregate formation. In order to avoid the formation of agglomerates, the dialysis technique, starting from a common solvent, is the recommended method for the micelle preparation. Notably, the nano-rings, an elaborate morphology, became available by surface induced self-assembly, by forming a thin film on specific surfaces, such as mica and silicone wafers. This procedure occurs through the solvent evaporation of the copolymer solution on the surface, which induces a “*frozen-in*” structure that is maintained after re-dissolution of the thin film [24]. From these studies, it turns out that, for a given copolymer sample, the self-association procedure has a major influence on its micellar characteristics, such as morphology, size, and size distribution.

It has to be noticed that some of these techniques may lead to kinetically “*frozen-in*” situations instead of a thermodynamic equilibrium between unimers and micelles, which are usually only observed when the core-forming polymer sequence has a low glass transition temperature (*T*_g_). As a general remark, it appears from the present literature survey that a strict distinction is not always made between kinetic “*frozen-in*” self-assembled systems and micelles in thermodynamic equilibrium with their unimers.

Finally, by the “crystallization-driven self-assembly” (CDSA) method, intensively studied by the groups of Winnik and Manners for poly(ferrocenylsilanes)-based copolymers, especially cylindrical micelles can be obtained from crystallizable block copolymers having a crystalline core block that is much smaller than the corona sequence [25,26,27].

### 2.4. Characterization Techniques

The critical micellar concentration (CMC), as well as the critical micellization temperature (CMT), are fundamental characteristics of copolymers solutions in a unimer/micelle thermodynamic equilibrium. CMC values are usually accessible by different fluorescence techniques [28,29], and to a minor extent, by surface tension measurements. Moreover, it has to be noted that the CMC values determined for “*frozen-in*” systems might be questionable. The micellar hydrodynamic diameter, *D*_h_, is determined by dynamic light scattering (DLS), transmission electron microscopy (TEM), and SAXS [30]. The micellar morphologies can be observed with different electron microscopy techniques, such as TEM and scanning electron microscopy (SEM). In particular, cryo-TEM and tomography has been applied by different authors [31,32,33] for the direct visualization of micellar nano-structures. In addition to these well-described size and morphology characterization techniques, other elaborate methods are available for the study of block copolymers self-assembly in organic media, such as super-resolution fluorescence microscopy technique, which is especially used for the visualization of cylindrical micelles obtained from crystallizable copolymers by the CDSA method [34].

## 3. AB and ABA Linear Block Copolymers in Organic Solvents

Within this overview of block copolymer self-assembly in organic solvents, the logical approach was to first consider the simplest molecular architectures of AB and ABA copolymers. This section provides a review of the micellar characteristics during the self-assembly of AB and ABA copolymers in non-polar and polar solvents, including their mixtures and ionic liquids (ILs), as well as in biocompatible solvents. This self-assembly is further described for AB copolymers, including crystallizable sequences.

**Figure 1:**
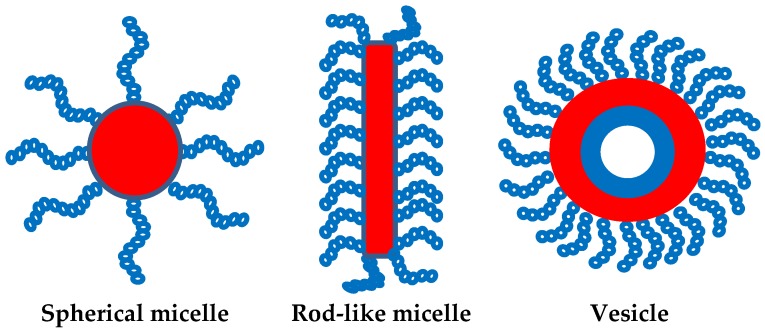
Basic micellar morphologies for AB copolymers.

The basic micellar morphologies correspond to spheres, cylinders, and vesicles, depending on the molecular characteristics of the copolymers, the selective solvent, and the micellization conditions. Figure 1 is an illustration of these basic morphologies.

**Figure 2:**
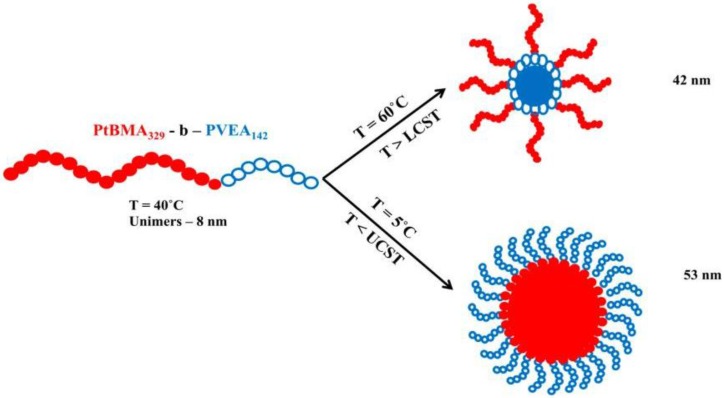
Micellization characteristics of PtBMA_329_-*b*-PVEA_142_ copolymer in methanol as a function of temperature (lower critical solution temperature (LCST) = 53 °C; upper critical solution temperature (UCST) = 32 °C). Adapted from Li et al. [35].

It has to be recalled that a given amphiphilic AB copolymer in a selective solvent of either the A or B block leads to two types of morphologies having either an A or B micellar core, respectively. This micellar structure inversion can also be created by thermal treatment, as demonstrated by Li et al. [35] for the self-assembly of poly(tert-butylmethacrylate)-*b*-poly[*N*-(4-vinylbenzyl)-*N*,*N*-diethylamine] (PtBMA-*b*-PVEA) copolymers in methanol. This possibility is schematically outlined in Figure 2:

### 3.1. Self-Assembly of AB and ABA Block Copolymers in Non-Polar Selective Solvents

Micellization of well-defined AB block copolymers, such as poly(butadiene)-*b*-poly(styrene) (PB-*b*-PS) and poly(isoprene)-*b*-poly(styrene) (PI-*b*-PS), has been studied quite extensively in the second half of the 20th century [1,7,36,37,38,39]. From the relatively few papers published on this topic since then, the objectives of the authors were mainly to examine specific aspects of the self-assembly of AB diblock copolymers in non-aqueous media. An interesting example of this type of study was that of Sotiriou et al. [37]. For a series of PS-*b*-PI diblock copolymers, tagged with a ω-lithium sulfonate (SO_3_Li) end group on either the PS or PI sequence, these authors examined the micellization behavior in *n*-decane as a function of the localization of the polar end-group. A micellar solution was obtained by direct dissolution and heating the block copolymer samples in *n*-decane. The principal micellar characteristics of these systems at 25 °C are provided in Table 2.

These results prove that the position of the SO_3_Li end-group has a considerable influence on the micellar characteristics. The SO_3_Li end-group fixed on the PS sequence, which forms the micellar core, leads to an increase in both *N*_agg_ and *R*_h_, with respect to the unlabeled sample. This effect may be attributed to the dipolar interaction of the SO_3_Li groups in a non-aqueous medium. For the SO_3_Li end-groups on the PI sequence, this interaction occurs in the solvent phase between the micelles as a result of the formation of polydisperse and interconnected SO_3_Li large aggregates with the PS cores.

Later on, Cheng et al. [40] studied the morphological changes of non-functionalized PS-*b*-PI copolymer in *n*-decane as a function of temperature and pressure using small-angle neutron spectroscopy (SANS). According to the authors, the increase in pressure from 200 to 16,000 psi, at room temperature, had no effect on the micellar characteristics (*N*_agg_, *R*_g_, and *R*_core_), but led to the formation of micellar agglomerates. At 60 °C and high pressure, the micelles underwent a macro-phase separation with the formation of sheet-like aggregates. The authors indicated that these morphological changes are attributed to the decrease of the “*n-decane quality*” for the PI sequences.

Growney et al. [41] examined the self-assembly of poly(styrene)-*b*-poly(ethylene propylene) (PS-*b*-PEP) diblock copolymers that are obtained by the hydrogenation of the PI sequence of PS-*b*-PI copolymers. As selective non-polar solvents of PEP, either *n*-heptane (δ = 15.2 MPa^1/2^) or *n*-dodecane (δ = 16.0 MPa^1/2^) was used, and star-like micelles with an *R*_h_ of around 40 nm were obtained in both of the solvents. The self-association of another type of PI-based diblock copolymer, such as poly(ethylene oxide)-*b*-poly(isoprene) (PEO-*b*-PI), was investigated by Bartels et al. [42] in *n*-decane. In this case, the micellar solutions were obtained using a precipitation method starting from a homogeneous copolymer solution in tetrahydrofuran (THF).

Wang et al. [43] observed by DLS a bimodal distribution attributed to unimers and spherical micelles for the self-assembly of a series of poly(styrene)-*b*-poly(2-vinyl pyridine) (PS-*b*-P2VP) diblock copolymers in the presence of a pure non-polar solvent, such as toluene. Moreover, these authors investigated the micellar aggregate formation by mixing different architectures of this type of copolymers, such as linear diblocks and triblocks, as well as branched star-like copolymers.

Finally, Arai et al. [44] studiedthe self-aggregation behavior of PS-based diblock copolymers in chloroform and 1,2-dichloroethane (DCE), using DLS and SLS. The micellar characteristics a series of poly(styrene)-*b*-poly[(ar-vinylbenzyl)trimethylammonium chloride] (PS-*b*-PV) diblock copolymers, obtained by RAFT polymerization, were directly correlated with the number average polymerization degree (*DP*_n_) ratio of the two blocks. For example, star-like micelles were observed for a ratio *DP*_n_(PS)/*DP*_n_(PV) >6, whereas brush-like micelles were obtained if this ratio was smaller than 6.

### 3.2. Self-Assembly of AB and ABA Block Copolymers in Polar Selective Solvents

In this section, the recent micellization studies completed for AB and ABA block copolymers in different pure polar solvents are summarized (Table 3).

From Table 3, it appears that the interest of the authors was focused on the (meth)acrylic- and vinyl ester-based copolymers that might have practical industrial application possibilities. Furthermore, it can be noticed that solvents having a solubility parameter in the range of 18 to 30 MPa^1/2^ (see Table 1), and in particular methanol, are suitable selective solvents.

### 3.3. Self-Assembly of AB and ABA Block Copolymers in Organic Solvent Mixtures

An alternative to pure organic solvents is provided by the mixtures of two solvents, which may allow for a gradual variation of the “*solvent quality*”. Sophisticated morphologies of micellar nano-particles become accessible using this typical approach.

The influence of the “*solvent quality*” on micellar characteristics was studied by Cho et al. [52] for a poly(styrene)-*b*-P4VP:poly(4-vinyl pyridine) (PS_400_-*b*-P4VP_167_) diblock copolymer sample in pure THF, THF/water, and THF/ethanol mixtures, respectively. The *R*_h_ of the micelles, with a P4VP core and PS corona, was around 23 nm in pure THF, significantly decreased to 14.1 nm for a solvent mixture composition of 95/5 *v*/*v*% THF/EtOH. Moreover, in the presence of ethanol, which is a “*good solvent*” for the P4VP core, micellar size *polydispersity* increased, whereas the aggregation number decreased.

Zhou et al. [53] investigated the self-assembly of poly(styrene)-*b*-PHFBMA:poly(2,2,3,3,4,4,4-heptafluorobutyl methacrylate) (PS-*b*-PHFBMA) copolymers in a mixture of THF and ethyl acetate (EtOAc) at different volume ratios, such as 5:0; 4:1; 3:2; and, 0:5. For these mixtures, the authors calculated the solubility parameters in order to study their influence on the copolymer’s self-aggregation behavior. As observed with TEM, the micellar morphology changed from spheres to vesicles with the increase in EtOAc content. By DLS, it turns out that the average size of the micelles increased from 140 to 190 nm, 235 and 267 nm, respectively, as a function of EtOAc volume fraction. Moreover, it was demonstrated by these authors that the morphology and the size of the micelles was highly influenced by the temperature.

Wang et al. [54] studied the self-aggregation of a PS_64_-*b*-PEO_827_ diblock copolymer in a mixture of cyclohexane/1,4-dioxane (80/20 wt %), using DLS and TEM. For the preparation of the micellar solution, the copolymer was at first directly dissolved in 1,4-dioxane and then the cyclohexane was added very slowly. Spherical micelles with a *D*_h_ of 50 nm were obtained from this procedure. A micellar morphological transformation was observed, from spheres to cylinders and vesicles, by decreasing the temperature from 25 to 0 °C and then to −10 °C. This transformation from spheres to vesicles, attributed to the increasing interfacial energy between the solvent and the PEO core, was highly temperature dependent and accompanied by an increase in the *D*_h_ from 50 to 1680 nm.

In addition to DLS and TEM techniques, the micellar morphological modifications were confirmed with SAXS. In this context, Rao et al. [55] investigated the self-aggregation of a PS_481_-*b*-P2VP_157_ diblock copolymer in a mixture of dimethylformamide (DMF) and methanol in order to prepare micelle-functionalized silica particles. Core-shell spherical micelles, with a *R*_h_ value of 22.5 nm, were obtained by direct dissolution of the copolymer in DMF, as a common solvent, followed by a slow addition of methanol.

Choi et al. [56] studied the self-assembly of PS_404_-*b*-PEP_886_ diblock copolymers in a mixture of 1-phenyldodecane (δ = 17.4 MPa^1/2^)/squalane (δ = 16.6 MPa^1/2^). At 110 °C and a 50/50 ratio of 1-phenyldodecane/squalane, the micellar *R*_h_ was equal to 280 nm, which is much higher than the value of 40 nm determined by Growney et al. [41] for the PS_315_-*b*-PEP_1203_ micelles in *n*-heptane, at the same temperature. Due to this comparison, solvents, such as heptanes and *n*-decane with δ values of 15 to 16 MPa^1/2^ appear to be more selective for PEP than the 1-phenyldodecane/squalane mixture, with an average δ value of around 17 MPa^1/2^.

### 3.4. Self-Assembly of AB and ABA Block Copolymers in Ionic Liquids

Ionic liquids (ILs), which are a special class of polar solvents, are interesting for environmental reasons when compared to common volatile organic solvents. In addition, excellent chemical and thermal stability, wide liquid temperature ranges, and low toxicity are the most important typical properties of ionic liquids. Due to these specific properties, ILs have become efficient solvents for the synthesis of block copolymers using the PISA technique, as outlined in a recent review by Derry et al. [57]. Moreover, the self-assembly of block copolymers in ILs has led to the development of original micellar structures. In connection with this topic, the recent relevant publications are listed in Table 4.

In addition to Table 4, typical and detailed examples are given to highlight the correlation between the micellar characteristics and the major system parameters, such as the relative sequence length of the copolymers, and, in particular, the respective solvent/copolymer solubility parameter.

Simone and Lodge [66] studied, by cryo-TEM and DLS, the self-assembly of a series of three PS-*b*-PMMA block copolymers with different compositions in 1-butyl-3-methylimidazolium hexafluorophosphate [BMIM][PF6]. This ionic liquid, with a δ value of 30 MPa^1/2^, is a selective solvent for the PMMA block. The reduction of the PMMA content leads to a morphological transition from spherical to cylindrical micelles. More recently, for the same type of hydrophobic/hydrophobic PS-*b*-PMMA block copolymer, Mok et al. [67] investigated the effect of the composition on the C.M.C in 1-ethyl-3-methylimidazolium bis(trifluoromethylsulfonyl)imide [EMIM][TFSI], an IL with a δ value of 27 MPa^1/2^. A decrease in the C.M.C values from 0.40 to 0.078 wt % was observed when the *DP*_n_ of the PS-core increased from 29 to 106.

**Figure 3:**
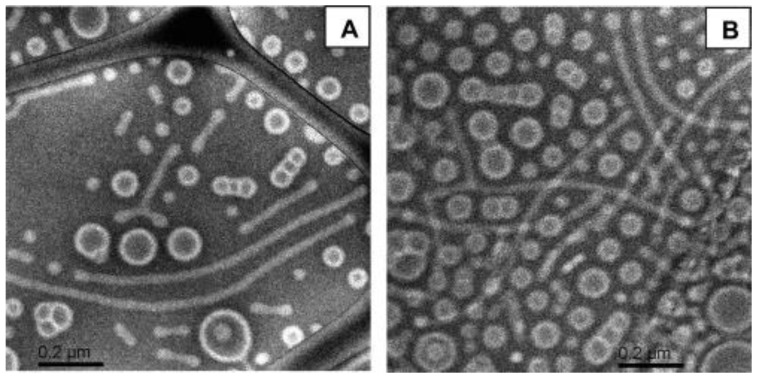
Cryo-TEM images of PB-*b*-PEO copolymer, with 0.25 mol % PEO, at a concentration of 1 wt % in [BMIM][PF6]. (**A**) Worm-like micelles with occasional Y-junctions and (**B**) micellar overlap. “Reprinted with permission from He, Y.; Li, Z.; Simone, P.; Lodge, T.P. Self-assembly of block copolymer micelles in an ionic liquid. *J. Am. Chem. Soc.* **2006**, *128*, 2745–2750. Copyright 2017 Americal Chemical Society”.

The micellization in [BMIM][PF6] ionic liquid of a series of hydrophobic/hydrophilic PB-*b*-PEO diblock copolymers, with fixed PB and increasing PEO sequence lengths, was investigated by He et al. [68]. By increasing the molar fraction of the PEO, micellar morphology evolved from worm-like micelles and bilayered vesicles to spheres. An illustration of these morphologies determined by cryo-TEM is provided in Figure 3.

More recently, the micellization of a quite similar PB-*b*-PEO copolymer series was investigated by Meli et al. [69] in [EMIM][TFSI] ionic liquid. These authors demonstrated that the direct dissolution of the copolymer led to the formation of large aggregates. However, a thermal treatment at 170 °C induced the formation of spherical micelles with *R*_h_ values of around 29 nm. For a given copolymer sample, it was of interest to compare the influence of the solubility parameter of both the PB-core and the solvent on the micellar characteristics. For this purpose, the authors studied the self-aggregation in a different IL, such as 1-butyl-3-methylimidazolium bis-(trifluoromethyl sulfonyl)imide [BMIM][TFSI], which has a slightly smaller δ value (26.7 MPa^1/2^ when compared to 27.6 MPa^1/2^ for [EMIM][TFSI]). Similar micellar characteristics were obtained for these two ILs. This experiment, performed with two different ILs with similar δ values, confirms that the δ value of the solvent is the key parameter in the macromolecular self-association process.

The self-assembly of ABA copolymers in ILs was predominantly investigated for PEO-*b*-PPO-*b*-PEO copolymers, also designated as Pluronics. Zhang et al. [70] determined the C.M.C values of three Pluronics with a constant PPO sequence length, such as L61 (PEO_3_-PPO_30_-PEO_3_), L64 (PEO_13_-PPO_30_-PEO_13_) and F68 (PEO_79_-PPO_30_-PEO_79_), in both 1-butyl-3-methylimidazolium tetrafluoroborate [BMIM][BF4] (δ = 24 MPa^1/2^) and [BMIM][PF6] (δ = 30 MPa^1/2^) ionic liquids. These authors found that the critical micellar concentrations increased as expected with the PEO sequence length. As an extension of these results, Lopes-Barron et al. [71] studied the self-association in deuterated ethylammonium nitrate (dEAN) of a similar series of Pluronics, such as F127 (PEO_106_-PO_70_-PEO_106_), P123 (PEO_20_-PPO_70_-PEO_20_) and L121 (PEO_5_-PPO_70_-PEO_5_). Pluronic samples with higher PEO/PPO molar ratios (F127 and P123) promoted the formation of spherical micelles, whereas small PEO/PPO ratios (L121) favor the formation of vesicles.

### 3.5. Self-Assembly of AB and ABA Block Copolymers in Biocompatible Organic Solvents

Among the above-mentioned non-aqueous block copolymer micellar systems, a large number may be considered as biocompatible. This especially occurs with block copolymer micelles in saturated aliphatic hydrocarbon solvents, such as *n*-decane, dodecane, etc. Another typical example of a non-aqueous self-assembly study was reported by Miller et al. [72] for poly(caprolactone)-*b*-poly(2-vinylpyrridine) (PCL-*b*-P2VP) copolymers in oleic acid, a biocompatible natural fatty acid. Spherical micelles, with a PCL core and an average size of 144 nm, were observed from the cryo-TEM images. Moreover, these authors investigated the loading of two model proteins into this micellar system.

Our research group studied the micellization of P2VP-*b*-PEO diblock copolymers in several biocompatible solvents, such as PEG400 (δ = 21.3 MPa^1/2^), paraffin oil (δ = 15.3 MPa^1/2^), and Miglyol 812 (δ = 17.3 MPa^1/2^) [73,74,75]. Spherical micelles, having a P2VP core and a *R*_h_ in the range of 23 to 25 nm were obtained by the self-assembly of P2VP_37_-*b*-PB_189_ copolymer sample in paraffin oil and Miglyol 812, which is a glycerine ester. However, micelles with a higher *R*_h_ of around 60 nm and a PB core, were formed in PEG400. The driving force for the self-assembly of these diblock copolymers is the polymer/solvent interaction parameter χ. Notably, block copolymer micellar systems based on natural oils, such as Miglyol 812, may be used for biomedical or cosmetic applications [73].

### 3.6. Crystallization-Induced Self-Assembly of AB and ABA Block Copolymers in Organic Solvents

Block copolymers that include a crystallizable sequence may lead to micelles having a partially crystallized core. This so-called “crystallizable-driven self-assembly” (CDSA) method was reviewed by different authors [76,77], and more recently, by Tritschler et al. [27]. This process, involving phase separation above the melting temperature (*T*_m_) and crystallization upon cooling, leads to a partially crystallized micellar core that is stabilized by the soluble sequence of the copolymer. By using this method, the groups of Winnik and Manners have obtained sophisticated and precise rod-coil micellar structures in organic solvents with well-controlled dimensions [27,28,29]. The width and the shape of these non-spherical micelles could be modified by varying the *DP*_n_ of the crystalline micellar core, the composition of the corona or the experimental conditions.

Anexample of crystallizable PB_54_-*b*-PEO_61_ block copolymer was studied by Mihut et al. [78] in *n*-heptane, which is a “*good solvent*” for PB. At 70 °C micelles are formed with a PEO core and PB corona, with an *R*_h_ of 12 nm. Upon cooling to 20 °C, a size increase to 140 nm was observed by DLS as a direct consequence of the PEO core crystallization. A similar example of crystallization-induced self-aggregation concerns the micellar solutions of poly(methylene)-*b*-poly(acrylic acid) (PM-*b*-PAA) block copolymer in DMF, which was reported by Wang et al. [79]. At 80 °C, the copolymer is molecularly dispersed in DMF. On cooling, the PM block crystallizes and self-aggregates into well-defined disk-like structures. In a further recent example, another research group [80] has studied the self-assembly of P2VP-*b*-PEO in a *n*-amyl acetate (δ = 17.4 MPa^1/2^)/*n*-butanol (δ = 23.1 MPa^1/2^) mixture. At 35 °C, these authors observed the formation of spherical micellar morphologies having a partially crystallizable PEO corona with a size of around 200 nm. With a fast temperature decrease, well-defined single crystals were obtained by crystallization of the PEO block. Recently, the groups of Winnik and Manners [81] studied the self-assembly of two amphiphilic crystalline-coil polyferrocenyldimethylsilane-*b*-poly(*N*-isopropylacrylamide) (PFS-*b*-PNIPAM) diblock copolymers in methanol, ethanol, and 2-propanol. Spherical micelles were formed in methanol and ethanol for the PFS_56_-*b*-PNIPAM_190_ sample whereas a mixture of spherical and cylindrical structures were noticed for this sample in 2-propanol. This behavior, which was also observed for PFS-*b*-P2VP copolymers, was probably due to the different solubility of the PFS in these solvents.

### 3.7. Concluding Remarks

To make conclusions about the literature survey concerning the self-assembly of polyA-*b*-polyB diblock copolymers, the aim of the present section is to provide a critical analysis of the published results. Moreover, our intention is to highlight the recent advances and trends that were observed in different steps of the self-assembly process of diblock copolymers in organic solvents.

For AB and ABA diblock copolymers, the recent investigations were focused on the synthesis of “*well-defined*” samples, with low *polydispersity* indices in composition and molecular weight. Although sequential anionic polymerization remains a favorite and well adapted synthesis technique, the so-called “*controlled free radical*” methods (RAFT, ATRP, NMP …) are now used to a large extent. In fact, these recent techniques have the advantage to provide access to a broader range of block copolymer types, in particular to those based on polar monomers. Among the recent synthesis trends, mention has to be made for the PISA process, which involves, in the polymerization step, the formation of a micellar system in organic solvent. A further synthesis trends concerns the preparation of end-functionalized and fluorescent labeled AB diblock copolymers. With respect to the determination of the *molecular characteristics*, NMR and SEC remain the “*classical*” analytic techniques. Unfortunately, quite a number of studies are published with indication of the “*equivalent PS*” number and weight average molecular weights of their products. Precise and actual molecular weight values of the copolymer sample would be accessible by SEC and simultaneous determination of the intrinsic viscosity [η]. Such multi-detector SEC devices with so-called “*universal calibration*” are presently standard equipments. In addition to SEC technique, diffusion ordered spectroscopy (DOSY), recently reviewed by Groves [82], could be a valuable tool to detect polyA and/or polyB homopolymer “*impurities*” in a synthesized polyA-*b*-polyB block copolymer.

As already previously mentioned in Section 2, most of the published micellar systems may be considered from a thermodynamic point of view as non-equilibrium systems between unimers and micelles. The characteristics, such as size and morphology of these “*frozen-in*” nanoparticles will depend to a large extend on their preparation procedure. The experimental conditions may in fact vary from a simple dissolution of the sample to an elaborated precipitation procedure in a selective solvent of either the polyA or polyB block of the copolymer. It has to be recalled that the most usual procedure consists in the solubilization of the copolymer in a common solvent. To this molecular dispersion is then added drop-wise the selective solvent of either the A or B block and the common solvent is then eliminated, in general, by dialysis, in order to end up with micelles dispersed in a pure selective solvent.

**Figure 4:**
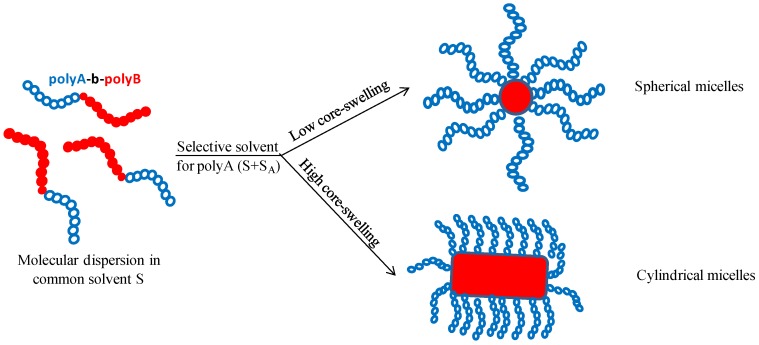
Schematization of the polyA-polyB diblock copolymer self-assembly process in solvents mixture.

Among the recent trends in *self-assembly techniques*, mention has to be made of the preparation procedure involving *solvents mixtures*, either mixtures of two organic solvents or of an organic solvent and water. This type of approach has the advantage that the solubility parameter and the solvent/polymer interaction parameter χ may be triggered step by step as a function of the volume fraction of the two solvents in presence. From the studies concerning the *self-assembly in organic solvents mixtures*, mentioned in Section 3.3, it could be noticed that these mixtures have a strong influence on particle size and morphology. A first type of approach, illustrated by Zhou et al. [53], is to generate a solvent mixture, having a solubility parameter δ_mix_, adapted as a selective solvent for either the A of B block of the AB copolymer. In a first approximation, it may be assumed that δ_mix_ = ø_1_δ_1_ + ø_2_δ_2_, with ø_1_ and ø_2_ the volume fractions and δ_1_, δ_2_ the solubility parameters of the two solvents. The major limitation of this concept is that both solvents have to be selective solvents of either the A of the B block. If this requirement is not met, then a partition of the solvents may occur, which leads to a swelling of the micellar core by either one or both solvents in presence. A second possibility to use organic solvent mixtures in the self-assembly process of polyA-*b*-polyB copolymers was investigated by different authors [54,55,56]. Their approach consists in the solubilization of the copolymer in a common solvent ***S***, a “*good*” solvent for both polyA and polyB sequences. To this molecularly dispersed copolymer is then added a given amount of selective solvent, such as for instance a solvent ***S_A_*** selective for the polyA block. This procedure leads to the precipitation of the polyB block and to the formation of micelles having a polyB core more or less swelled by the common solvent ***S***, as schematically illustrated in Figure 4. These micelles are stabilized by the polyA sequence solubilized in the ***S*** + ***S_A_*** solvent mixture. The presence of the common solvent ***S*** in the final micellar system represents the major difference with respect to the “*classical*” systems, where the common solvent is eliminated, in general, by dialysis, in the final preparation stage.

For polyA-*b*-polyB micellar systems in a selective solvent it is now well established that the spherical, cylindrical, and vesicular morphology is mainly determined by the relative volume fractions of the core and corona. Spherical morphologies, for instance, are in general generated when *DP*_A_, the polymerization degree of the polyA sequence, is low with respect to *DP*_B_ (the corona forming block). In the case of solvent mixtures, the relative volume fractions of core and corona are mainly determined by their swelling characteristics as a function of the solvent compositions and temperature. At a given temperature, spherical micelles have a tendency to be formed at a lower swelling degree of the micellar core. At this point, there is still a lack of information concerning: (i) the onset of self-assembly as a function of the molecular characteristics of the copolymer; (ii) the evaluation of the copolymer conformation with increasing selective solvent concentration; (iii) the partition of common and selective solvents in the micellar core and corona; and, (iiii) the evolution of the swelling degree of the core as a function of the volume fraction of the solvents. Regarding the *self-assembly in solvents mixtures*, it is established that this process opens interesting perspectives for the development of new morphologies and it could also provide an important insight in the “*classical*” self-assembly process itself.

In our opinion, the scattering and electron microscopy for *micellar characterization techniques* are well documented in the literature. As a minor point, it might be suggested that the DLS characterizations should be carried out as a function of concentration, with extrapolation to zero concentration. The determination of the *interphase*, the dimensions of the transition zone between core and corona, could be of interest for specific applications, such as those where micellar systems are used as nanoreactors. Finally, it is quite surprising that in contrast to water-based micellar systems, only very few results were published concerning the CMC and CMT values of block copolymers in organic solvents. A similar remark can be made for the determination of the aggregation number *N*_agg_, the average number of polymer chains per micelles.

The “*crystallization-induced self-assembly*” topic, including rod-coil and other micellar structures, was very recently reviewed by Tritschler et al. [27]. This self-assembly method offers the possibility to prepare non-spherical micellar morphologies with controlled dimensions having promising applications in various domains.

## 4. Self-Assembly of Linear ABC Triblock Terpolymers in Organic Solvents

Linear ABC triblock terpolymers have opened an extensive research area for the development of sophisticated micellar structures over the last decade. Up to now, several excellent review papers have been published on this topic [2,7,83]. For the self-assembly of ABC copolymers it turns out once again that a majority of the studies were focused on aqueous-based micellar systems and only relatively few of them on the micellization in organic media. Wyman and Liu [83], for instance, showed the fascinating morphologies that are becoming available for a given ABC copolymer by precise control of the micellization conditions, including the solvent mixtures. More recently, Gröschel and Müller [84] extended this review by providing a detailed insight of the multi-compartment nanostructures that are accessible, as well with AB and ABC triblock terpolymers.

**Figure 5:**
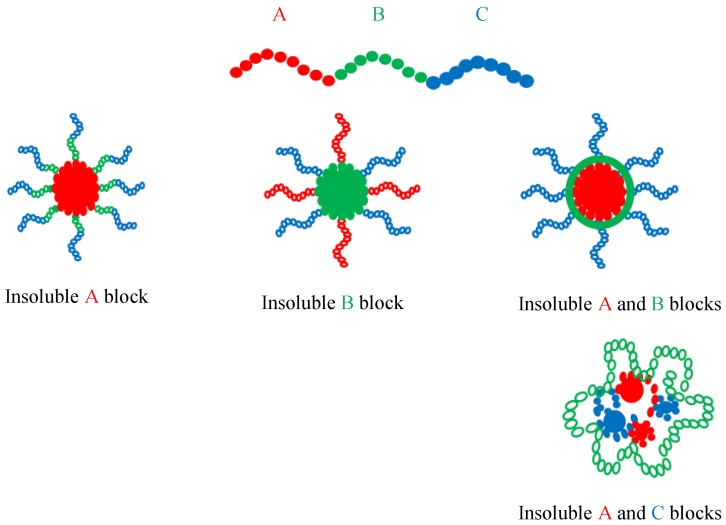
Basic micellar morphologies for linear ABC triblock terpolymers.

The objective of this chapter is to highlight the recent developments, including the present author’s contributions, of ABC triblock terpolymer micellar systems generated in organic solvents. Polar and non-polar solvents will be taken into consideration, as well as mixtures of organic solvents. According to Wyman and Liu [83], the basic micellar spherical morphologies of ABC triblock terpolymers are outlined in Figure 5, as a function of the solvent selectivity.

The sequence organization, as schematically displayed in Figure 5 for spherical micelles, is similar for cylindrical (worm-like) and vesicle micellar morphologies.

**Figure 6:**
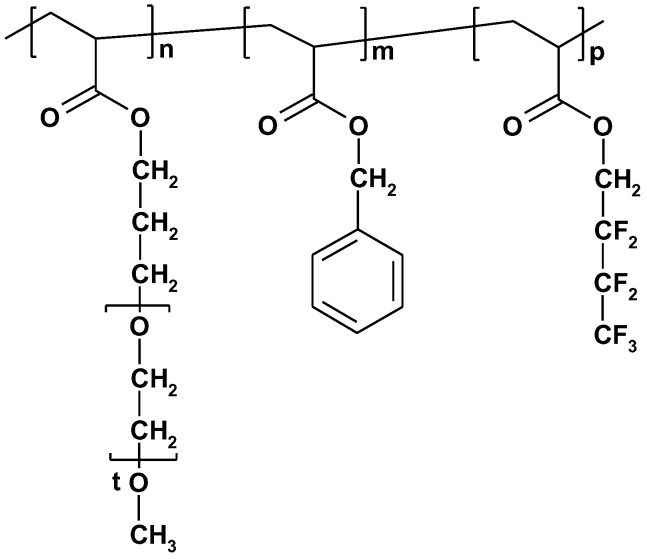
Chemical structure of the ABC triblock terpolymer POEGA-PBzA-PFA [85].

For ABC-type linear triblock terpolymers, the sequence arrangement is an additional parameter that must be considered with respect to diblock copolymers. In fact, for a given ABC sample in selective solvent conditions, the micellar characteristics are different from the corresponding ACB and BAC structures. Up to now, the effect of the sequence arrangement was demonstrated for the self-assembly of triblock terpolymers in aqueous media [85,86] or in water/organic solvent mixtures [87]. Marsat et al. [85] examined the micellar formation in aqueous medium of an ABC, ACB, and BAC triblock terpolymer comprising a hydrophilic, a hydrophobic, and a fluorophilic sequence, such as poly[oligo(ethylene oxide) monomethyl ether acrylate]-poly(benzyl acrylate)-poly(1H,1H-perfluorobutyl acrylate) (POEGA-PBzA-PFA). The chemical structure of this ABC copolymer is shown in Figure 6.

Analogous to the previous chapter, the present one will be organized in a similar way by considering the self-assembly of ABC triblock terpolymers in non-polar, polar, and solvent mixtures. This means that a given ABC copolymer may appear in different subsections.

### 4.1. Self-Assembly of Linear ABC Triblock Terpolymers in Non-Polar Selective Solvents

A typical example of self-assembly in non-polar solvent has been studied in our research group for a series of PB-P2VP-PEO triblock terpolymers in *n*-heptane, a selective solvent of the PB sequence, as well as in aqueous medium [7,88,89].

**Figure 7:**
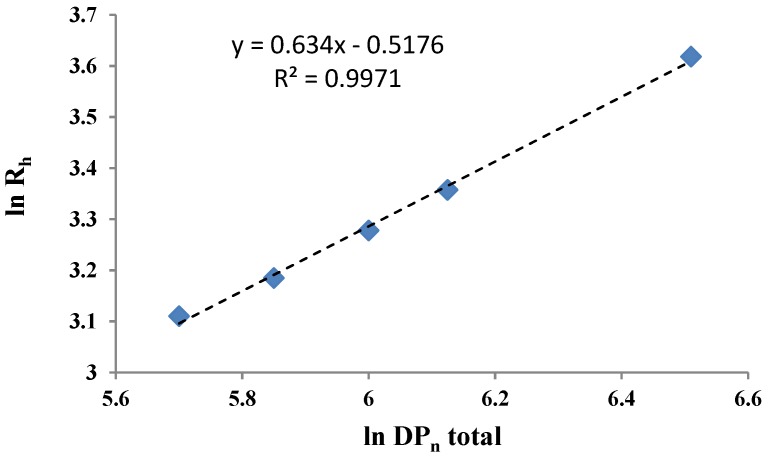
Logarithmic variation of the *R*_h_ as a function of *DP*_n_ total of PB-P2VP-PEO triblock terpolymers in *n*-heptane [88].

This type of ABC copolymers has been selected as the solubility parameter of the sequences is well differentiated, such as 17, 21, and 20.8 MPa^1/2^ for the PB, P2VP, and PEO, respectively. Moreover, the P2VP sequence is easily protonated or quaternized, yielding a pH- and electrolyte-sensitive water-soluble block. Figure 7 illustrates the variation of the micellar particle size as a function of the total *DP*_n_.

From Figure 7, it can be noticed that R_h_ scales as *DP*_n_^0.64^ in reasonable agreement with the exponent of 0.68 predicted by Noolandi and Hong [90] for diblock copolymers. In a simplified approach, the *R*_h_ values could further be correlated with the individual *DP*_n_ values of the copolymers [88]. From a practical point of view, it was demonstrated that PB-P2VP-PEO copolymers with a P2VP middle block are efficient dispersing and stabilizing agents for TiO_2_ pigments in non-aqueous as well as in aqueous media [89]. Another example of the self-assembly studies of P2VP-based copolymers investigated in our research group was the micellization of poly(2-vinyl pyridine)-poly(tert-butyl methacrylate)-poly(cyclohexyl methacrylate) (P2VP-PtBMA-PCHMA) triblock terpolymers in methylcyclohexane, which are of practical interest as “viscosity improvers in motor-oil formulations” [7].

The self-assembly of P2VP-based triblock terpolymers, such as PS-P2VP-PMMA, was also studied by Tsitsilianis and Sfika [91] in toluene, a selective solvent for the PS and PMMA sequences. These authors found that both the *N*_agg_ and *R*_h_ of spherical core-shell micelles, having a P2VP-core and a corona of PS and PMMA, depend on the *DP*_n_ of the insoluble P2VP sequence. Core-shell micelles, based on amino (meth)acrylates, were also obtained by Bütün et al. [92] for poly[2-(diisopropylamino)ethyl methacrylate]-poly[2-(dimethylamino)ethyl methacrylate]-poly[2-(*N*-morpholino)ethyl methacrylate] (PDPA-PDMA-PMEMA) triblock terpolymers in hexane. In this case, the PDMA and PMEMA sequences are in the core of the micelles, which have a *R*_h_ of 46 nm, whereas the PDPA block formed the solvated corona.

### 4.2. Self-Assembly of Linear ABC Triblock Terpolymers in Polar Selective Solvents

The recent publications concerning the self-assembly of linear ABC triblock terpolymers in polar solvents are summarized in Table 5.

From Table 5, it may be noticed that the recent investigations concerning the self-assembly in polar solvents were mainly focused on alcohols, such as methanol, which is a selective solvent of the end-blocks. This implies that the middle block preferentially forms the micellar core.

### 4.3. Self-Assembly of Linear ABC Triblock Terpolymers in Organic Solvent Mixtures

The self-assembly of ABC terpolymers in organic solvent mixtures was performed essentially through the precipitation method starting from a homogeneous solution in a common solvent for all three blocks, followed by the addition of a non-solvent. As previously described, Bütün et al. [92] studied the self-assembly of two PDPA-PDMA-PMEMA triblock terpolymers, not only in *n*-hexane, but also in a mixture of CHCl_3_/*n*-hexane, starting from a unimer’s solution in CHCl_3_. In this case, core-shell-corona micelles were obtained with a PMEMA core and a *R*_h_ value of 15 nm.

**Figure 8:**
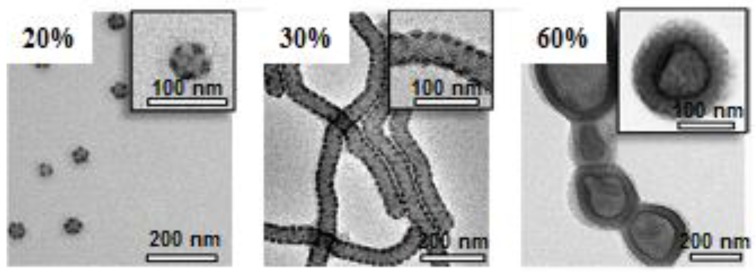
Micellar morphological changes of PS-PB-PMMA triblock terpolymers in acetone/isopropanol mixtures as a function of isopropanol content. “Reprinted with permission from Löbling, T.I.; Ikkala, O.; Gröschel, A.H.; Müller, A.H.E. Controlling multicompartment morphologies using solvent conditions and chemical modification. *ACS Macro Lett.* **2016**, *5*, 1044–1048. Copyright 2017 Americal Chemical Society”.

The morphology of core-shell-corona structures, formed by the self-assembly of PS-P4VP-PEO triblock terpolymers in DMF/ethanol mixtures, was reported by Wang et al. [99] as a function of temperature. A further interesting study concerning the morphological behavior as a function of the “*solvent quality*” was performed by Löbling et al. [97,100] for a series of PS-PB-PMMA and PS-PB-PtBMA triblock terpolymers in acetone/isopropanol mixtures. The micellar morphology modifications determined by cryo-TEM as a function of the isopropanol content are illustrated in Figure 8.

More recently, Cong et al. [98] completed a similar study for PI-PS-P2VP triblock terpolymers in THF/ethanol mixtures.

### 4.4. Miscellaneous Self-Assembly Studies of Linear ABC Triblock Terpolymers

Given this context, and in analogy to AB diblock copolymers, studies have been completed on the behavior of ABC self-assembly in ionic liquids, the influence of partially crystallized moieties, and the development of biocompatible or thermo-sensitive systems.

Self-assembly of AB diblock copolymers in ILs has been studied extensively, as shown in Section 3. However, it seems that up to now ABC triblock terpolymers have attracted less attention. Mention could be made only of the publication of Kitazawa et al. [101]. These authors investigated the morphological behavior of a poly(benzyl methacrylate)-poly(methyl methacrylate)-poly(2-phenylethyl methacrylate) (PBnMA-PMMA-PPhEtMA) triblock terpolymer, as a function of temperature in 1-ethyl-3-methylimidazolium bis(trifluoromethanesulfonyl)amide [C2MIM][NTF2] (δ ~ 26 MPa^1/2^) at high copolymer concentrations, such as 10 and 20 wt %.

The groups of Winnik and Manners have extended the CDSA method from AB diblock copolymers to ABC triblock terpolymers by studying the morphology of a series of well-defined coil-crystalline-coil poly(ferrocenylphenylphosphine)-poly(ferrocenyldimethylsilane)-poly (dimethylsiloxane) (PFP-*b*-PFS-*b*-PDMS) terpolymers in hexane, which is a selective solvent for PDMS [102]. Cylindrical micelles were obtained for the sample having the *DP*_n_(PFP) <6, whereas spherical micelles were observed if the *DP*_n_(PFP) = 11. The longer PFP chains hinder the crystallization of the PFS sequence leading thus to the formation of spherical morphologies. More recently, the crystallization-induced self-assembly of poly(styrene)-poly(ethylene)-poly(methyl methacrylate) (PS-PE-PMMA) triblock terpolymer in toluene and dioxane, a good and bad solvent for the semicrystalline PE, respectively, was investigated by Schmelz et al. [103]. As a function of the “*solvent quality*”, either spherical or worm-like crystalline-core micelles were observed by these authors.

The self-assembly of ABC triblock terpolymers in biocompatible non-aqueous solvents was investigated in our research group for a PB-P2VP-PEO sample. At low concentrations in PEG400, this triblock terpolymer forms spherical micelles with a PB-core and P2VP/PEO corona. An interesting feature appeared at concentrations higher than 3 wt %, at which a thermo-reversible gel, due to the formation of H–bonds, was noticed for these micellar systems [104].

### 4.5. Concluding Remarks

It is undeniable that ABC triblock terpolymers have opened an important research area, in particular for the development of sophisticated micellar morphologies. This topic having recently be reviewed in detail, the aim of the present section is to highlight more specific aspects related to the synthesis and the self-assembly of ABC linear triblock terpolymers. A very large range of this type of copolymers has been prepared up to now. In our opinion, it would be of interest for further developments of the field to synthesize homologous series of ABC, BCA, and BAC structures in order to complete the demonstration that the sequence distribution has a major influence on the micellar characteristics. For this type of copolymers, it would further worthwhile to check in different selective solvents the CMC, CMT values, as well as the *N*_agg_, which are scarcely reported up to now.

In the same manner as for AB diblock copolymers, the self-assembly of ABC copolymers in *solvent mixtures* could contribute to the understanding of the micellization process. This type of studies may be carried-out in specific solvent mixtures, as already demonstrated by different authors [85,90,97]. In order to complete the results reported by Zhang et al. [87] and by Bethausen et al. [105], it could be of interest to examine in detail the self-assembly of ABC terpolymers in solvent/water mixtures. Of further interest will be the self-assembly studies of ABC terpolymers in ionic liquids and biocompatible organic solvents, which are only very scarcely discussed in the literature.

Crystallization of ABC terpolymers in bulk, thin films and on surface, such as two-dimensional (2D) has been examined in detail over the last years. However, in comparison with AB diblock copolymers only a few studies are available concerning the CDSA method for ABC terpolymers.

## 5. Self-Assembly of AB and ABC Graft Copolymers in Organic Solvents

Analogous to AB block copolymers, the AB graft copolymers may develop the basic sphere, cylinder, and vesicle micellar morphologies, as a function of molecular characteristics, selective solvent type, copolymer concentration, and self-aggregation process. Micellization of synthetic- and polysaccharide-based graft copolymers in aqueous media has been reviewed very recently by the present authors [23]. Therefore, it was necessary to complete this topic by providing an overview on the self-assembly of various types of graft copolymers in organic solvents. After recalling some basic graft copolymer characteristics, this section focus on AB graft copolymers, including the concept of unimolecular micelle formation, followed by the morphologies of ABC graft structures.

**Figure 9:**
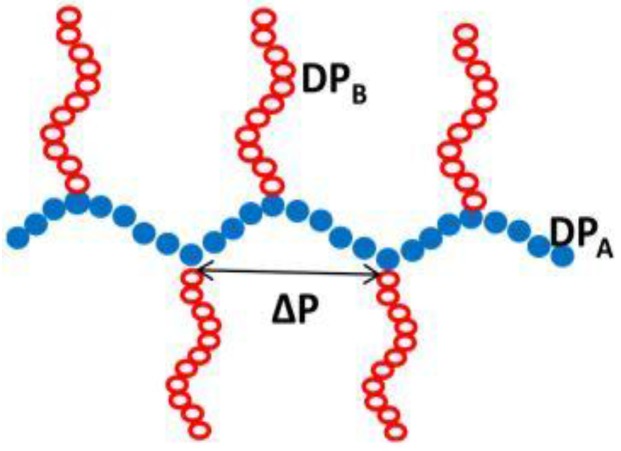
Schematically representation of an AB graft copolymer.

As schematically shown in Figure 9, a graft copolymer is essentially defined by the *DP*_A_ and *DP*_B_ of the backbone and graft chain, respectively. A major molecular characteristic is further the graft density, which represents the number of side chains per 100 backbone monomer units. Moreover, assuming that the graft chains are randomly distributed along the backbone, the average distance Δ*P* between two grafting sites is thus accessible.

Already at the end of the last century, Kikuchi and Nose [106] clearly demonstrated that graft copolymers have a greater tendency than the corresponding block copolymers to form unimolecular micelles by intramolecular association of the backbone sequences. This tendency was well illustrated for PMMA-*g*-PS in organic solvents, such as iso-amyl acetate or in the presence of a mixture of acetonitrile and acetoacid ether. It appeared that the tendency of forming unimolecular micelles increases with an increasing graft density and molecular weight of the grafted chains. At high graft densities, unimolecular micelles are formed by intramolecular collapse of the backbone chain. In a theoretical approach, Borisov and Zhulina [107] admitted that the collapsing of the backbone chain occurs with formation of a “*pearl neck-lace*” stabilized by steric repulsion of the soluble side chains.

From the literature survey, as already mentioned, it turned out that the number of publications related to self-assembly of graft copolymers in aqueous media increased over the last two decades due to the predominant applications possibilities of these aqueous systems. Purely organic solvent-based systems have become rather scarce. In the following section, an overview of the recent investigations concerning the self-assembly of synthetic and natural-based graft copolymers in pure organic solvents, are reviewed.

### 5.1. Self-Assembly of AB Graft Copolymers

For the self-assembly studies of polysaccharide-based graft copolymers in organic solvents, a special mention could be made of the investigation of Liu et al. [108]. These authors reported the synthesis of ethyl cellulose-*g*-poly(acrylic acid) (EC-*g*-PAA), which leads to unimolecular micelles in DMF, methanol, and their mixtures with water by increasing the graft density. Francis et al. [109] described the synthesis of chitosan-*g*-PS and prepared micellar dispersions in DMF as these systems are of interest as metal complexing agents.

Analogous to the peptide/PtBMA water and organo-soluble composite investigations by Saha et al. [110], Bose et al. [111] reported peptide-based graft copolymers, such as tyrosine-*g*-polyoxazoline. The micellar structures of this type of copolymer were examined in aqueous and non-aqueous media. Concerning the recent example of a purely synthetic graft copolymer, Stepánek et al. [112] investigated the micellization in methanol and water of poly(4-methylstyrene)-*g*-poly(methacrylic acid) (P4MS-*g*-PMAA). In methanol, a tendency to form unimolecular micelles was demonstrated.

PtBMA-based graft copolymers, such as PS-*g*-PtBMA, were reported by Gromadzki et al. [113]. These authors examined the conformation of this type of copolymers in a non-selective solvent, such as a THF, as well as in n-amylalcohol, a selective solvent of the PtBMA graft chains. By increasing the graft density, a stretching of the PS backbone was noticed for the unimolecular micelles.

A typical example of fluorinated graft copolymers was studied by Koda et al. [114]. This type of thermo-sensitive copolymer leads to micelle formation in both fluorinated solvents and in water. As a final and recent example from this category of graft copolymers, mention has to be made of poly(phenyl carbodiimide)-*g*-poly(styrene) (PCD-*g*-PS) self-assembly in methanol described by Kurilov et al. [24]. The nano-ring morphologies obtained for this rod-coil copolymer were generated by surface-induced self-assembly in thin films, prepared by the controlled evaporation of THF/methanol copolymer solutions.

### 5.2. Self-Assembly of ABC Graft Copolymers

Various structures are available for ABC three-component graft copolymers and their micellization characteristics in aqueous media have been recently reviewed [23]. Concerning the micelle formation in organic media, a typical example that is related to the self-assembly of ABC graft copolymers, such as poly(1-dodecene-co-pMS)-*g*-PEO, was published by Liu et al. [115]. These authors performed an extensive study of their micellization behavior in *n*-octanol. As a function of the molecular weight of the PEG, 350, 750 and 2000 g·mol^−1^, respectively, the C.M.C increased from 1.28 × 10^−4^ to 1.95 × 10^−4^ g·mL^−1^. For the reversed spherical micelles, the size distributions were relatively large with typical diameters in the range of 71 to 186 nm.

**Figure 10:**
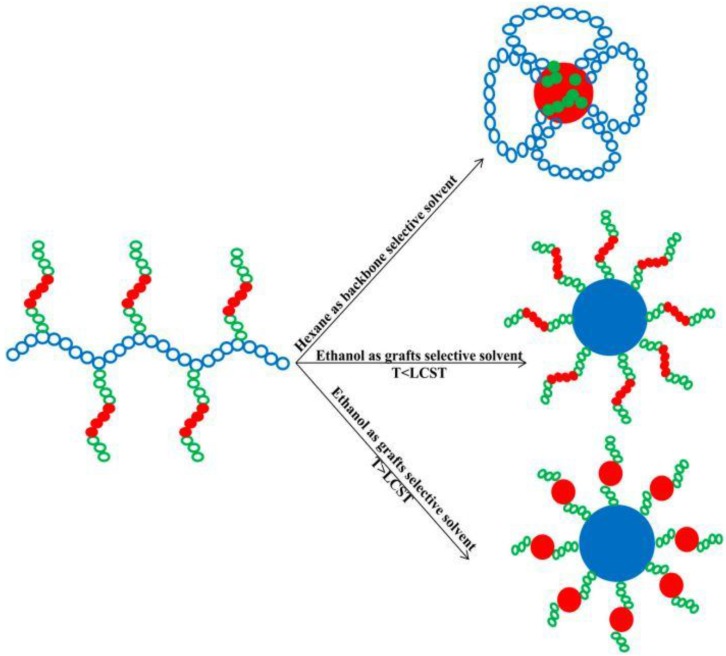
Micellar conformations of PI-*g*-Pluronic copolymers in hexane (backbone selective solvent) and in ethanol (grafts selective solvent). Adapted from Alexander et al. [116].

A detailed micellization study by small angle neutron scattering (SANS) was carried out by Alexander et al. [116] for PI-*g*-Pluronics. These authors investigated the micellization behavior of these graft copolymers in different solvents, such as THF, a common solvent for PI and Pluronics, and hexane and ethanol, which are selective solvents for PI and Pluronics, respectively. As expected, “*flower-like*” micelles were obtained in hexane, the selective solvent for the PI backbone chain, whereas “*star-like*” micelles were formed in ethanol. These morphologies are illustrated in Figure 10. This study was completed by the determination of the micellization onset as a function of the copolymer concentration in THF/hexane mixtures, ranging from pure THF to pure hexane.

**Figure 11:**
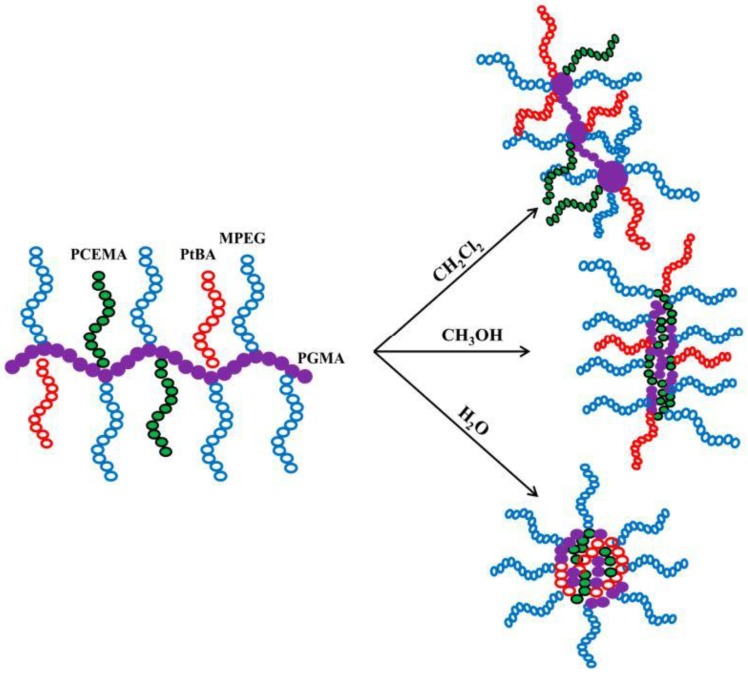
Micellar conformation of PGMA-*g*-(PCEMA-PtBA-MPEG) copolymers in CH_2_Cl_2_, CH_3_OH and H_2_O selective solvents, respectively. Adapted from Mo et al. [117].

Mo et al. [117] extended the self-assembly concept to multigraft copolymers. Using the “*click chemistry*” preparation technique, they synthesized a series of PGMA-*g*-(PCEMA-PtBA-MPEG) having the same P(GMA-N_3_) backbone, and three different type of side chains. In DMF, a common “*good solvent*” for both the backbone and side-chains, they observed by AFM a stretched “*worm-like*” conformation. With three different selective solvents (CH_2_Cl_2_, CH_3_OH, and H_2_O), the morphologies of the unimolecular micelles varied from a “*neck-lace*” to “*worm-like*” and to multi-component spherical structures, as schematically illustrated in Figure 11.

### 5.3. Concluding Remarks

With the present synthesis methods, outlined in our recent review article [23], a wide range of AB and ABC graft copolymers with various architectures are available in order to examine their behavior in organic solvents. In fact, over the last decade, this topic has attracted less research interest than self-assembly studies in aqueous media. With respect to block copolymers, graft copolymers have the advantage to form *unimolecular micelles* by adjusting the graft density. Moreover, they provide also access to natural-based products, such as polysaccharides comprising structures. By taking into account these specific features, original developments may be expected in this research area.

## 6. General Conclusions and Perspectives

The self-assembly of block and graft copolymers in aqueous media is undeniably at the present a major research trend given their biomedical application possibilities. Nevertheless, from our overview it turns out that micellar systems in non-aqueous solvents are of ongoing theoretical and practical interest.

For AB and ABA block copolymers, which are considered as “*model molecular architectures*”, the recent studies were mainly focused on their self-assembly in polar and non-polar solvents, as well as in solvent mixtures. This last approach has the advantage that the micellar characteristics, such as size and morphology, can be triggered in a continuous way by adjusting the solvent mixture composition, and thus the “*solvent quality*”. Over these last years, a major interest was further devoted to the micellization in ionic liquids as selective solvents. As this research area has reached its maturity, further developments may be expected for polyA-*b*-poly(B-*co*-C) copolymers with a random or gradient B and C blocks. This approach is in fact an alternative to adjust the solubility parameter.

ABC triblock terpolymers have developed into an extensive research area for the study of sophisticated micellar morphologies. Substantial advances were reported, in particular, for aqueous and non-aqueous systems, yielding multi-compartment micellar structures. For these multi-compartment nanoparticles, it was demonstrated that the sequence arrangement of the ABC triblock terpolymers is a major structural parameter. Different morphologies are obtained in a given selective solvent for ABC, BAC, and BCA of the same composition and molecular weight. Even if the challenge remains for the polymer chemist, this concept should be estended to ABC, BAC, and BCA copolymers to self-aggregation in organic solvents, including the biocompatible ILs.

Despite their wide-spread application possibilities, AB and ABC graft copolymers have attracted up-to-now relatively minor interest concerning the micellization in organic solvents. With respect to block copolymers, graft copolymers have the advantage that by adjusting the graft density brush-like structures, and unimolecular micelles can be obtained. Graft copolymers, based on natural polymeric precursors, may create opportunities for self-assemblies in organic solvents.

Finally, as well for block copolymers as for graft copolymers, an efficient control of the molecular architectures and the self-assembly parameters are still a major challenge for further investigations in this research area.

## Figures and Tables

**Table 1:** Solubility parameters (δ) of the most usual non-polar and polar solvents.

Non-Polar Solvents	δ (MPa^1/2^)	Polar Solvents	δ (MPa^1/2^)
*n*-Hexane	14.9	Ethyl acetate	18.1
*n*-Heptane	15.2	Dichloromethane	19.8
*n*-Decane	15.8	Acetone	19.9
Hexadecane	16.3	THF	19.5–20.2
Cyclohexane	16.7	Isopropanol	23.6
Toluene	18.2	*n*-Propanol	24.4
Chloroform	18.7	Acetonitrile	24.4
1,4-Dioxane	19.9–20.5	DMF	24.9
		DMSO	26.5
		Ethanol	26.5
		Methanol	29.7
		Water	47.9
		Ionic liquids	24.0–32.0

**Table 2:** Micellar characteristics for the PS-*b*-PI diblock copolymers in *n*-decane at 25 °C as determined by Sotiriou et al. [37].

Sample	Nagg	Rh (nm)
PS_60_-*b*-PI_203_	10.2	10.4
(SO_3_Li)-PS_60_-*b*-PI_203_	23.4	14.2
PS_55_-*b*-PI_151_-(SO_3_Li)	17.0	660.0 ^a^
	11.0 ^b^	75.0 ^b^

^a^ large *polydispersity*. ^b^ values at 30 °C.

**Table 3:** Micellization studies of AB block copolymers in pure polar solvents.

AB Block Copolymers	Selective Solvent for A Block	Selective Solvent for B Block	Micellar Characteristics	Ref.
PS_48_-*b*-PNVP_99_	-	CH_3_–OH	*R*_h_ = 16 nm *R*_g_/*R*_h_ = 0.65 C.M.C. = 0.13 mg·mL^−1^ *N*_agg_ = 10	[45]
POSS-PMMA_144_-*b*-P(MA-POSS)_2.6;9.6;11.3_	THF	-	*R*_h_ = 85; 148; 80 nm Core-shell micelles	[46]
PMMA_340;400_-*b*-PtBMA_134_	-	2-ethylhexanol	*R*_h_ = 19.4 ÷ 28.2 nm Spheres and cylinders	[47]
PSAMA_15_-*b*-P(Boc-Phe-HEMA)_7;17;37;75_	-	DMF; DMSO; ACN	*R*_h(DMF)_ = 119 ÷ 318 nm *R*_h(DMSO)_ = 37÷90 nm *R*_h(ACN)_ = 24 ÷ 48 nm Spheres	[48]
PEtOx_10_-*b*-PNBA_7;17;31;48_	-	CH_2_Cl_2_	*R*_h_ = 45 ÷ 60 nm Spheres	[49]
PVAc_57_-*b*-(PFHE-stat-PVAc)_95_	CH_3_–OH	-	*R*_h_ = 10 ÷ 40 nm Spheres	[50]
PMMA_41;54;73_-*b*-PsfMA_59;46;27_	THF; ACN; CHCl_3_	-	*N*_agg(THF)_ = 8 *N*_agg(CHCl3)_ = 26 *N*_agg(ACN)_ = 410 *R*_h(THF)_ = 60–70 nm Spheres	[51]

The copolymers are designated by PX*_m_*_,*n*_-PY*_m_*_′,*n*′_, where *m*, *n*, *m*′ and *n*′ are the *DP*_n_ values. PNVP: poly(*N*-vinylpyrrolidone); POSS-PMMA: polyhedral oligomeric silsesquioxane-poly(methyl methacrylate); PSAMA: poly(2-(methacryloyloxy)ethyl stearate); P(Boc-Phe-HEMA): poly(tert-butyloxycarbonyl phenylalanine methacryloyloxyethyl ester); PEtOx: poly(2-ethyl-2-oxazoline); PNBA: poly(2-nitrobenzyl acrylate); PVAc: poly(vinyl acetate); PFHE: poly(perfluorohexylethylene); PsfMA: poly(1H,1H,2H,2H-perfluorodecyl methacrylate).

**Table 4:** Self-assembly studies of AB block copolymers in pure ionic liquids.

AB Block Copolymers	Selective Solvent for A Block	Selective Solvents for B Block	Micellar Characteristics	Ref.
PEO_432_-*b*-PNIPAM_106_	[BMIM][BF4] δ = 24 MPa^1/2^	-	*R*_h_ = 25 nm L.C.M.T = 200 °C U.C.M.T = 60 °C	[58]
PS_529;548;981_-*b*-P2VP_543;543;923_	-	[BMIM][CF3SO3] δ = 25 MPa^1/2^	Spherical micelles	[59]
PEO_341_-*b*-P(AzoMA-*r*-NIPAM)_177_	[C4MIM][PF6] δ = 23 MPa^1/2^	-	*R*_h_ ~ 120 nm	[60]
PEO_432_-*b*-PnBMA_99;183_	[BMIM][TFSI] δ = 26 MPa^1/2^ [EMIM][TFSI] δ = 27 MPa^1/2^	-	C.M.T = 120–150 °C	[61]
PEGE_109;113;104_-*b*-PEO_54;115;178_ PGPrE_98_-*b*-PEO_260_	PAN; EAN δ = 25–26 MPa^1/2^	-	spherical micelles for PEO_178_-*b*-PEGE_104_ Disk-shape micelles for PEO_54_-*b*-PEGE_109_	[62]
PEGE_104_-*b*-PEO_178_	[C4MIM][PF6] δ = 23 MPa^1/2^	-	*R*_h_ = 13 nm	[63]
PMMA_250_-*b*-PnBMA_92;169;218;246;310;373;549_	[EMIM][TFSI] [BMIM][TFSI]	-	*R*_h_ = 17.8 ÷ 34.6 nm *R*_core_ = 5.8 ÷ 23.2 nm *N*_agg_ = 41 ÷ 432	[64,65]

L.C.M.T and U.C.M.T: lower and upper critical micellization temperature; PNIPAM: poly(*N*-isopropylacrylamide); P(AzoMA): poly(4-phenylazophenyl methacrylate); PEGE: poly(ethyl glycidyl ether); PGPrE: poly(glycidyl propyl ether); [BMIM][CF3SO3]: 1-butyl-3-methylimidazolium trifluoromethanesulfonate; [C4MIM][PF6]: 1-butyl-3-methylimidazolium hexafluorophosphate; EAN: ethylammonium nitrate; PAN: propylammonium nitrate.

**Table 5:** Self-assembly of linear ABC triblock terpolymers in polar solvents.

ABC Triblock Terpolymers	Selective Solvent for A Block	Selective Solvent for B Block	Selective Solvent for C Block	Micellar Characteristics	Ref.
PtBA_107_-*b*-PCEMA_193_-*b*-PGMA_115_	CH_3_–OH	-	CH_3_–OH	Vesicles and cylinders	[93]
PB_800_-*b*-P2VP_190_-*b*-PtBMA_380;550_	Acetone	-	-	*R*_h_ = 43; 44 nm *R*_g_ = 34; 36 nm *N*_agg_ = 203; 174	[94]
PtBA_110_-*b*-PCEMA_195_-*b*-PSGMA_115_	Propanol	-	-	Cylinders	[95]
PnBA_28_-*b*-PS_37_-*b*-P2VP_73_	-	-	CH_3_–OH	*R*_h_ = 27 nm Spheres	[96]
PS_306;510;516_-*b*-PB_151;258;140_-*b*-PMMA_340;260;76_	DMAc	-	DMAc	Spheres	[97]
PS_385_-*b*-PI_485_-*b*-P2VP_829_	-	-	C_2_H_5_–OH	Ellipsoid	[98]

PtBA: poly(tert-butyl acrylate); PCEMA: poly(2-cinnamoyloxyethyl methacrylate); PGMA: poly(glyceryl monomethacrylate); PSGMA: poly(sucinnated glyceryl monomethacrylate); PnBA: poly(*n*-butyl acrylate); DMAc: dimethylacetamide.
